# Evaluating the potential of gold, silver, and silica nanoparticles to saturate mononuclear phagocytic system tissues under repeat dosing conditions

**DOI:** 10.1186/s12989-017-0206-4

**Published:** 2017-07-17

**Authors:** James L. Weaver, Grainne A. Tobin, Taylor Ingle, Simona Bancos, David Stevens, Rodney Rouse, Kristina E. Howard, David Goodwin, Alan Knapton, Xiaohong Li, Katherine Shea, Sharron Stewart, Lin Xu, Peter L. Goering, Qin Zhang, Paul C. Howard, Jessie Collins, Saeed Khan, Kidon Sung, Katherine M. Tyner

**Affiliations:** 1 Center for Drug Evaluation and Research, Food and Drug Administration, Silver Spring, MD 20993 USA; 2 National Center for Toxicological Research, Food and Drug Administration, Jefferson, AR 72079 USA; 3 Center for Devices and Radiological Health, Food and Drug Administration, Silver Spring, MD 20993 USA

**Keywords:** Durable nanoparticles, repeat dose, gold nanoparticles, silver nanoparticles, silica nanoparticles

## Abstract

**Background:**

As nanoparticles (NPs) become more prevalent in the pharmaceutical industry, questions have arisen from both industry and regulatory stakeholders about the long term effects of these materials. This study was designed to evaluate whether gold (10 nm), silver (50 nm), or silica (10 nm) nanoparticles administered intravenously to mice for up to 8 weeks at doses known to be sub-toxic (non-toxic at single acute or repeat dosing levels) and clinically relevant could produce significant bioaccumulation in liver and spleen macrophages.

**Results:**

Repeated dosing with gold, silver, and silica nanoparticles did not saturate bioaccumulation in liver or spleen macrophages. While no toxicity was observed with gold and silver nanoparticles throughout the 8 week experiment, some effects including histopathological and serum chemistry changes were observed with silica nanoparticles starting at week 3. No major changes in the splenocyte population were observed during the study for any of the nanoparticles tested.

**Conclusions:**

The clinical impact of these changes is unclear but suggests that the mononuclear phagocytic system is able to handle repeated doses of nanoparticles.

**Electronic supplementary material:**

The online version of this article (doi:10.1186/s12989-017-0206-4) contains supplementary material, which is available to authorized users.

## Background

Nanotechnology is being applied across the medical industry in an effort to develop innovative, next generation drugs and devices with optimized performance and improved quality, safety, and efficacy [1]. Although many nanoparticles used for medical applications are designed to degrade within the body, there have been increasing amounts of durable nanoparticle products proposed for medical uses. Durable nanoparticles do not immediately dissolve into their molecular entities after administration (e.g. drug nanocrystals), or degrade through normal biodegradation mechanisms within the body (e.g. liposomes). Rather, durable nanoparticles remain in the particulate state during administration, distribution, accumulation, and, in some cases, elimination. For the purposes of this manuscript, durable nanoparticles also include particles with slow dissolution/degradation (e.g. will eventually break down within the body, but not at the rate where clearance would occur during a weekly dosing scheme). Such durable nanoparticles are seen in the Food and Drug Administration (FDA) Center for Drug Evaluation and Research (CDER) as drug delivery platforms and imaging agents. FDA’s Center for Devices and Radiological Health (CDRH) may see these particles as implant wear debris, internal sensors, device coatings, and integrated into wound dressings [2, 3].

Overwhelming numbers of in vivo studies show that durable NPs accumulate in the mononuclear phagocyte system (MPS), which is more prevalent in specific tissues (e.g. liver, spleen, lung and bone marrow) [4, 5]. Such accumulation is largely size independent and occurs both in the presence or absence of NP surface coatings (note that *which* tissues within the MPS that the NPs accumulate may vary by size and surface coatings) [6–8]. Accumulation in organs occurs primarily through macrophage uptake [9, 10]. Once in the MPS organs, the durable NPs remain there, or have protracted elimination [11, 12]. With such biopersistence, exposure to the NPs may last years and potentially span the lifetime of the patient. This exposure is especially important in the treatment of chronic diseases where therapies are administered repeatedly to the affected population, with the potential for increased accumulation over the course of treatment.

Although many of these biodistribution and toxicity studies have been completed for acute single dosing of nanoparticles, fewer studies have been completed for repeat dosing of these materials. In terms of pharmaceutical relevance, this absence of repeat-dosing leaves a significant knowledge gap as repeatedly dosing durable nanoparticles could lead to saturation of the MPS, leading to unanticipated toxicities or a different biodistribution profile than a single dose, even when dosed at a sub-toxic level. Such information would aid in the risk assessment of using durable nanoparticles in drug products or medical devices. The goal of the current research was to determine if repeated exposure to sub-toxic doses of three common types of durable nanoparticles could saturate the MPS of the liver and spleen, and/or cause different bioaccumulation and/or toxicity. Three durable nanoparticles were investigated: 10 nm gold nanoparticles (AuNPs), 10 nm silica nanoparticles (SiO_2_ NPs), and 50 nm silver nanoparticles (AgNPs).

## Methods

### Materials

SiO_2_ NPs (10 nm, Ludox, SM-30 colloidal silica) were purchased from Polysciences (Warrington, PA). Citrate-capped AuNPs (10 nm) were purchased from SPI Supplies (West Chester, PA). AgNPs (50 nm) were citrate capped, endotoxin free, silver seeded, and purchased from Nanocomposix (San Diego, CA). 5% dextrose in water (D5W) was used as received (Hospira, Lake Forest IL).

### NP stock solution characterization

Size, particle stability (agglomeration/aggregation) and zeta potential of NPs were measured as previously described [9, 13]. Briefly, SiO_2_ NPs (10 nm) were sterilized by filtration (using a 0.1 μm Millex filter unit); AuNPs (10 nm) were concentrated and filtered as previously described [9]. AgNPs were used as received. All NP stock solutions were fully characterized either as received or diluted in deionized water. The weight concentration in g/L for each lot of NPs stock solutions in water was initially determined by thermal gravimetric analysis (TGA) (TA Instruments). 25 μL of stock solution was placed in the TGA pan and heated from ambient temperature at a rate of 2 °C/min. For AuNPs and AgNPs, dry NP weights were read at 500 °C. For SiO_2_ NPs, weights were read at 200 °C. Stock solution dilutes were analyzed using neutron activation analysis (NAA analysis) (Au, Ag) and inductively coupled plasma mass spectrometry (ICP-MS) analysis (Au, Ag, Si). For ultraviolet–visible (UV–vis) analysis, AuNP and AgNP stock solutions were diluted 10 μL into 2000 μL di water and the spectra was collected at 400 nm to 750 nm at 2 nm intervals. AuNP concentration was determined though Beer’s Law at wavelengths of 450 nm (ε = 10.65 L/g^**.**^cm), 506 nm (ε = 14.18 L/g^**.**^cm) and 520 nm (ε = 15.0 L/g^**.**^cm) and the results averaged [14]. AgNP solutions were diluted 100 times and run at the UV–vis parameters listed above. Absorbance was read at 418 nm (ε = 179.2 L/g^**.**^ cm).

Dynamic light scattering (DLS) and Zeta potential of all 3 NPs were determined either in the as received solution or by diluting NPs in water 100–200 times. Samples were analyzed on a Malvern Nano ZS in pre-cleaned vials. Samples were run at 25 °C in triplicate with the dispersion solution viscosity and refractive index corrected for the dispersion medium. Z average and histograms for number and intensity weighted measurements were recorded. For zeta potential measurements, samples were run 3 times in water with automatic parameter selection. Transmission electron microscopy (TEM) analysis was completed for all 3 NPs. Aliquots of diluted NPs were air dried on holey carbon coated copper grids (Quantifoil, EMS, PA USA) and analyzed on a JEOL 1400 TEM at 80 kV. At least 5 fields of view were imaged and all discrete particles in the field of view were measured for size analysis (average of ~40 particles counted).

NPs were further characterized via DLS and TEM following dispersion into the dosing medium (D5W) at projected in-use concentrations to evaluate stability within the dosing solution. DLS histograms were obtained at 4–6 h, 24, and/or 72 h to evaluate NP stability. In addition, NPs were dispersed into rat serum (sourced in house from training animals) to further evaluate NP stability.

Proton induced X-ray emission (PIXE) analysis was completed through a contract with Elemental Analysis Inc. (Kentucky, USA) to confirm NP purity. Lack of endotoxin contamination was confirmed by Limulus Amebocytes Lysate (LAL) gel clot formation assay at a minimum concentration of the expected dosing solution concentration and according to the package insert (Associates of Cape Cod Inc).

### Animals

All experimental procedures were approved by the White Oak Institutional Animal Care and Use Committee and carried out in the White Oak Animal Facility of the US FDA (Silver Spring, MD, USA) in accordance with the Guide for the Care and Use of Laboratory Animals (National Research Council, 1996). Female Balb/C mice (Taconic) were 8 weeks old at the initiation of the experiments, microchipped, and housed in groups under standard environmental conditions with free access to water and rodent chow (PMI Nutrition).

### NP dosing solution preparation and characterization

Dosing solutions were prepared on the morning of dosing. Dosing solution concentrations were determined based upon the average weight of the animals to be dosed (calculated the day before) and by stock solution concentration as determined by TGA [14, 15]. All solutions were prepared in a biosafety cabinet using fresh, sterile D5W.

Dosing solutions were characterized post dosing with no further dilution by DLS and TEM. Zeta potential was performed only if there was sufficient dosing material remaining. pH of the dosing solution was checked via Hydrion Papers (1 --12). Aliquots of the dosing solutions were also analyzed with NAA (AuNP and AgNP) or ICPMS analysis (SiO_2_ NP).

### NP dosing

Mice were dosed once per week for up to eight weeks via intravenous administration of the tail vein. All animals were dosed with either NP colloidal suspension or control (D5W) at a volume of 200 μL. AuNPs were dosed at a concentration of 10 mg/kg. AgNPs were dosed at a concentration of 5 mg/kg. SiO_2_NPs were dosed at a concentration of 5 mg/kg. Any missed or partial doses as well as any gross observations (e.g. scarred tails or barbering) were reported as soon as they were noticed.

### Animal necropsy

Each week, a cohort of 11 animals (7 nanoparticle treated, 4 control treated) were euthanized via exsanguination under isofluorane (e.g. animals were terminated 1 week after the previous injection). Blood was collected in serum tubes via cardiac puncture and immediately spun down to collect serum. Brain, kidney, spleen, liver, lung, sternum, and tail were collected and weighed. The right kidney was scored transversely and placed in formalin for histopathology analysis. The left kidney was placed in an NAA vial or Eppendorf tube for elemental analysis. The spleen was weighed and then sectioned into thirds. One portion of the spleen was placed in Dulbecco’s Phosphate Buffer Solution (DPBS) on ice for splenocyte analysis by flow cytometry. The middle portion of the spleen was weighed and placed in a vial for elemental analysis. The final third was placed in formalin for histopathology analysis. For consistency, the same portion of the spleen was used for each analysis. The left lobe of the liver was scored and placed in formalin for the histopathology analysis. The remaining liver was weighed and placed in a vial for elemental analysis. The lung, tail, and sternum were weighed and stored for elemental analysis. Tails were also evaluated for obvious signs of doses missing the tail vein (discoloration of tail vein and/or scarring). Any gross observations during necropsy were noted by a trained investigator performing the observations blinded. A schematic of the dosing scheme is represented in Additional file 1: Figure S1.

Three to five additional animals were dosed with each NP to ensure that sufficient animals would be available at the end of 8 weeks for analysis (e.g. to account for mis-doses or animal death). At the end of 8 weeks, these 3–5 animals remained that had completed the 8 week dosing regimen. These animals were necropsied along with 4 animals that had been used for injection training (with D5W, no NP injections). The following list of tissues were collected, weighed, and placed into individual vials: blood, brain, kidney, spleen, liver, heart, lung, thymus, sternum, uterus/ovaries, a portion of skin from the mammary region, stomach/intestines/lymph nodes/pancreas, muscle (biceps femoris), and tail. The remainder of the carcass was also stored in a vial after removal of the microchip. Tissues were allowed to dry prior to capping.

### Serum chemistry

Serum was stored at −80 °C until use. 100 μl of serum from each animal was run on an Abaxis VetScan Classic Instrument using a Comprehensive Diagnostic Profile rotor (Abaxis, California). The fourteen parameters measured were albumin, alkaline phosphatase, alanine aminotransferase, amylase, creatinine, sodium, total protein, total bilirubin, blood urea nitrogen, calcium, phosphorus, glucose, potassium, and globulin.

10 μl of serum was analyzed using the MesoScale Discovery (MSD, Maryland) 96 well Multi-Array Pro-Inflammatory-7Ultra-Sensitive Kit on a MSD Sector Imager 2400 following the instructions included in the kit. Using the standard curve provided with kit, the concentrations of the following seven analytes were determined: INF-γ, IL-1β, IL-10, IL-12p70, IL-6, KC/GRO/CINC, and TNF-α.

### Splenocyte phenotyping

Spleen sections were processed through a cell strainer into a 50 mL conical tube. The strainer was rinsed with 6 mL DPBS, and the rinse placed into the conical tube. Strainers were then incubated with Liberase (1.6 U/mL)/DNase (100 μg/mL) at 37 °C for 15 min in a 60 mm Petrie dish. Cell strainers were rinsed with an additional 6 mL of flow buffer (DPBS containing 0.5% BSA, 1% Penicillin/Streptomycin and 1% Citrate-Dextrose-phosphate) and the rinse placed into the conical tube. Cells were centrifuged at 300 g for 5 min at room temperature. The supernatant liquid was carefully aspirated and the pellet re-suspended in 1 mL RBC lysis buffer (BioLegend). Cells were incubated at room temperature for 5 min prior to centrifugation at 300 g for 5 min. Supernatant was carefully aspirated and the pellet re-suspended in 1 mL of flow buffer. 5 μL of cell solution was diluted into 10 mL IsoFlow™ Sheath Fluid (Beckman Coulter) and the cells counted via Coulter Counter. Based upon the cell count, 500,000 cells were added to each flow tube. Eleven flow tubes were prepared (10 antibodies and 1 no cell control), and blocked with 1 μL blocking buffer (TruStain fcX™ (anti-mouse CD16/32) antibody from BioLegend) for 5 min at room temperature. A stock master mix containing all the antibodies was prepared as listed in Additional file 1: Table S1 and 6.5 μL antibody stock added to each cell suspension. Cells were incubated at 4 °C for 20 min and then washed with 600 μL of flow buffer via 5 min of centrifugation at 300 g. The supernatant liquid was carefully aspirated and the pellet re-suspended in 250 μL flow buffer. Measurements were conducted on a FACS Aria III flow cytometer with 100,000 events being measured for each sample. A full compensation matrix was performed prior to analysis. For analysis, cells were initially gated on forward versus side scatter (SSC) and intact cells were then gated to include all CD45+ cells. CD3 and CD19 positive cells were directly measured from the CD45+ population. The T-cell subsets CD4 and CD8 were measured from the CD45 and CD3+ population. Monocytes and neutrophils were measured on CD11b versus Ly6G where strongly double positive cells are counted as neutrophils. Monocytes were the Ly6G negative but CD11b positive population.

### Histopathology

Portions of the liver, spleen, and kidney were sent to Experimental Pathology Laboratories, Inc. (Sterling, VA) for trimming, embedding, staining with H & E and microscopic evaluation under an existing contract with the US FDA. Non-gradable lesions, such as neoplasms, were indicated as present. Microscopic findings were correlated to gross observations in histopathology incidence tables with the following grading system:

1. = minimal
2. = slight/mild
3. = moderate
4. = moderately severe
5. = severe/high

Additional file 1: Tables S2, S3, S4 contain additional information on the parameters evaluated.

### NAA

NAA was used for analysis of stock solutions, dosing solutions, and tissues samples for Au and Ag. Sample vials were allowed to dry prior to capping the vials to prevent leakage during sample transfer and analysis. NAA analysis was performed at Becquerel Laboratory (Ontario, Canada) using their standard operation procedure SOP (BQ-NAA-4, Elemental Analysis via INAA) through a contract with Elemental Analysis Inc. (Kentucky, USA). Gold and silver values were reported as total gold in μg/vial.

### ICP-MS

ICP-MS was used for analysis of stock solutions and dosing solutions for Au, Ag, and Si, and for tissue samples for Si. SiO_2_ NPs stock solutions were stored in darkness at room temperature (RT). SiO_2_ NPs were vortexed briefly then sonicated for 15 min prior to characterization. Aliquots were digested using CEM Discover SP-D digestion system with 35 mL Teflon liners and Pyrex vessels with Nitrogen flow for cooling. Aliquots of known volumes (<0.5 mL) were digested using 2.5 mL of hydrofluoric acid and 0.5 mL of nitric acid (Fisher trace metal grade). Teflon magnetic stirring bars were used in each sample at a medium controlled stir speed. Vessel temperature was increased from RT to 185 °C with a 5 min ramp and held for 2 min at a pressure of 300 psi with power setting of 300 within the organic settings of the system. AuNP stock solutions were stored in darkness at RT following concentration of the manufacture’s original stock solution. Immediately prior to characterization, AuNPs were briefly vortexed then sonicated for 5 min. A measured volume (<0.5 mL) was transferred to Teflon vessels and digested with 2.5 mL of nitric acid and 0.5 mL of hydrochloric acid (Fisher trace metal grade) using a Mars Express Microwave System with a ramp to 200 °C at 10 min with a 20 min hold time and 100% power and appropriate wattage according to carousel capacity. AgNP stock solutions were stored at 4 °C in darkness. AgNP samples were placed in darkness at RT for 30 min prior to characterization then briefly vortexed and sonicated for 5 min. Aliquots of known volumes (<0.5 mL) were transferred to Teflon vessels and digested with 3 mL of nitric acid (Fisher trace metal grade) using a Mars Express Microwave System using the same protocol as described above for the AuNPs. Silica NPs (Nanocomposix 20 nm amino and non-functional Si NPs), silver NPs (Nanocomposix) and gold NPs (NIST 60 nm 8013) were used as quality controls and digested along with the samples using the same method parameters. Si (Inorganic Ventures/Lot:E2-S103010 and Ultra Scientific/Lot:R00037), Ag (SCP Science), and Au (SCP Science) ionic salt solutions were also prepared in the same manner and used for quality controls for the non-microwave digested standards used to generate the standard curve as well as to ensure the optimal performance of the instrument and the analytical method being applied.

Following microwave digestion, samples were diluted to 15 or 50 mL total volume with water (18 Ω Ultrapure Millipore® in house system) and indium was added as an internal standard (PlasmaCal/Lot:S140303008). A standard curve was generated using non-microwave digested Si (Inorganic Ventures/Lot:E2S103010 and Ultra Scientific/Lot:R00037), Au (PlasmaCal/Lot:S131204002) or Ag (PlasmaCal/Lot:S131107006) ionic salts at 0, 25, 50, 75, 100, 250, 500 and 750 ng/mL and contained Rh and In (final sample concentration 300 ng/mL). Metal analysis was conducted for Ag and Au using an Agilent 7700 Series ICP-MS. Metal analysis for SiO_2_ was conducted on a Perkin Elmer Nexion D Series ICP-MS fitted with an organic sample intake kit. Data was transferred to and analysis was completed using Excel for all elemental analysis.

### Tissue analysis:

Metal analysis for Si was perfomed using ICP-MS. Tissues were harvested and stored at −80 °C until analysis. Tissue weight was recorded when harvested and again prior to digestion to ensure sample integrity (note that no weight differences were noted during the course of the study). Tissue samples were microwave digested as described above according to the nanoparticle of exposure. Rhodium was again used a quality control standard and added prior to digestion. Following microwave digestion, a known volume was aliquoted and diluted to 50 mL with water and internal standard indium was added (final sample concentration 300 ng/mL). SiO_2_ NPs and Si ionic salts were also prepared and analyzed concurrently as quality control standards with Si associated tissue samples. Ag, Au and Si were quantified using standard curves of 0, 25, 50, 75, 100, 250, 500 and 750 ng/mL standards. Standard matrix matching was achieved by addition of digested certified reference material DOLT-4 Dogfish liver for gold and silver standards. Silica standard matrix matching was achieved using digested rodent tissue that was previously analyzed to determine Si concentration. Metal analysis was conducted using a Perkin Elmer Nexion D Series ICP-MS fitted with an organic sample intake kit. Data was transferred to and analysis was completed using Excel for all elemental analysis.

#### Statistical analyses

Statistical analysis was performed using two-way ANOVA followed by Tukey’s multiple comparison tests (serum, cytokines) to compare across treatment groups. NP treated versus untreated organ weights were analyzed through a T test with the Holm-Sidak method for multiple comparisons (a = 0.05) and *p* < 0.05 for significance for each dosing point.

## Results

### NP Characterization

**Fig. 1:**
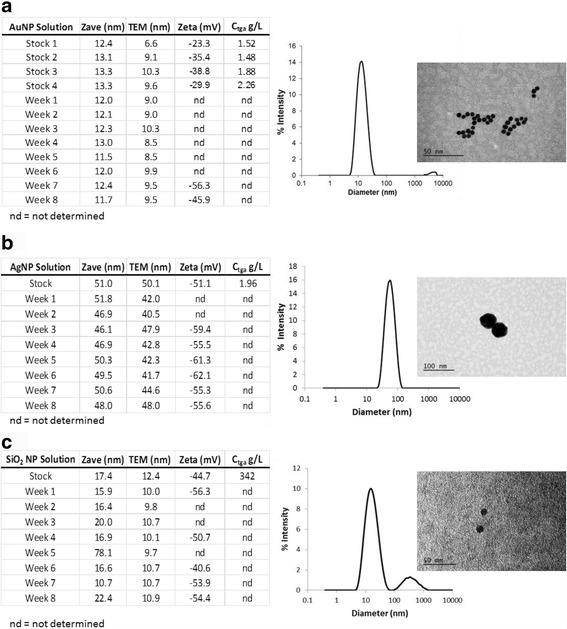
Representative DLS and TEM characterization of NP stock solutions and summary of characterization for stock and dosing solutions. Tables on the *left* reflect the characterization summary for each stock and dosing lot of NP solutions. Histograms and micrographs on the *right* show representative results from the dosing solutions. **a** AuNPs scale bar = 50 nm. **b** AgNPs scale bar = 100 nm. **c** SiO_2_ NPs scale bar = 50 nm. Note the agglomeration present in AuNPs and SiO_2_ NP DLS histograms as indicated by the smaller peaks at larger diameters

No NP stock solution tested above the detection level for endotoxin (0.25 EU/mL). Figure 1 shows representative DLS histograms and TEM images for the three NP stock solutions as well as a summary of size, zeta potential and concentration for stock and dosing solutions, where applicable. All NPs exhibited a roughly spherical morphology. AuNPs had a Zave of ~12 nm and a TEM average of ~9 nm. AgNPs had a Zave of ~49 nm and a TEM ave. of ~44 nm. SiO_2_ NPs had a Zave of ~24 nm and a TEM ave. of ~11 nm. Both AuNPs and SiO_2_ NPs indicated some agglomeration as evidenced by DLS (Fig. 1a, c, respectively). This agglomeration impacted the DLS Zave measurement for SiO_2_ NPs, as indicated by the larger Zave as compared to the TEM measurement, and in particular for week 5. AuNP and AgNP size characterization are in line with expected particle diameters as indicated by the suppliers. Purity of the stock solutions was evaluated through PIXE analysis. AuNP stock solution showed the presence of Au and Cl. SiO_2_ NPs showed the presence of Si and Cl as well as Fe, Zr, and Zn in trace amounts (63 ppm, 44 ppm, and 2 ppm, respectively). AgNPs could not be analyzed due to agglomeration and settling of the samples prior to measurement.

Dilution into the dosing solution of D5W did not significantly impact the stability of the NP solutions as indicated by DLS histograms and TEM images, the summary of which may be found in the tables of Fig. 1. Time within the dosing solution did not significantly impact NP stability as indicated by DLS analysis (Additional file 1: Figure S2). All dosing solutions were used within 3.5 h of preparation. Stability evaluation of the NPs within rat serum was confounded due to the protein signal arising from the serum (data not shown).

### Gross observations, histopathological observations, and blood chemistry following repeat dosing

#### AuNP

Mice were dosed once per week for up to 8 weeks with 10 mg/kg AuNP based upon stock solution concentrations as measured by TGA. During the course of the study, one mouse dosed with AuNPs was euthanized early due to extreme lethargy at week 3. Upon necropsy, it was observed that the mouse had an enlarged, discolored spleen. The liver was also noted to be enlarged, discolored, and mottled in all lobes. Histopathology on the tissues later determined that the mouse had a myelogenous leukemia of granulocytic lineage. The presence of the leukemia is considered to be incidental and unrelated to treatment. The mouse was subsequently removed from all analyses. No other significant incidence of gross toxicity was noted during the experiment.

**Fig. 2:**
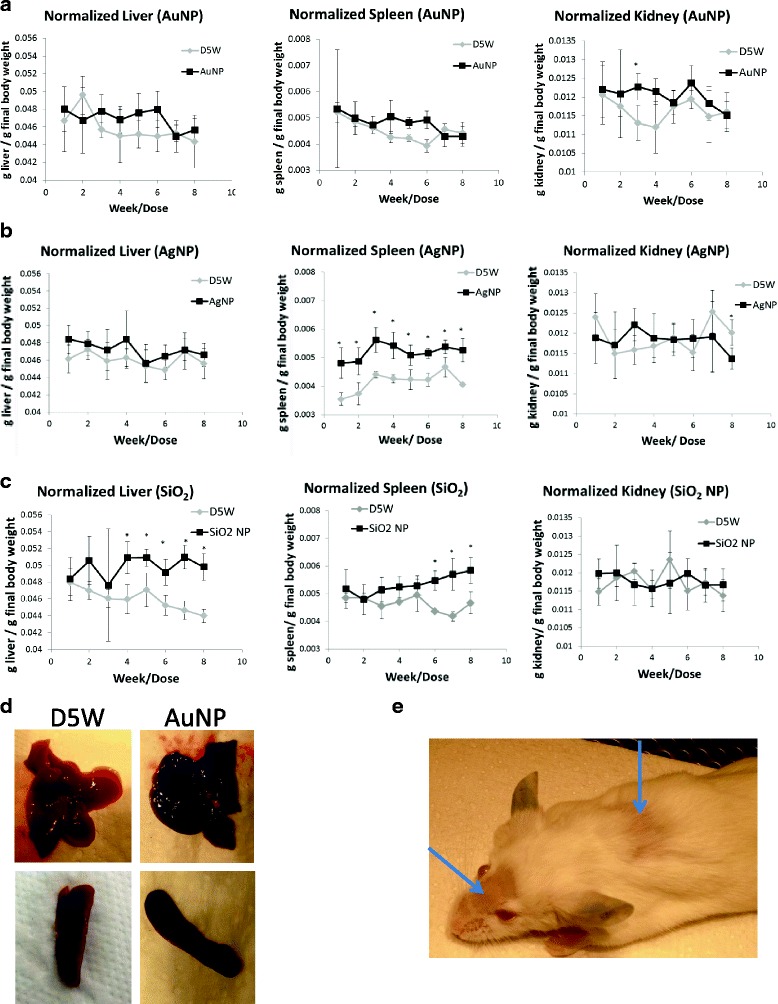
Normalized organ weights of liver, spleen, and kidney for mice exposed to (**a**) AuNPs (**b**) AgNPs (**c**) SiO_2_ NPs. As mice were still growing during the course of the dosing regimen, organ weights are normalized to final body weight (g organ/g final body weight). Error bars are standard deviation (**d**) Representative images of the liver and spleen after 8 weeks of dosing of either D5W (*left*) or AuNPs (*right*). AuNP treated tissues were dark and discolored throughout (**e**) Representative image of SiO_2_ NP treated animal exhibiting barbering. *Arrows point* out barbered areas

No major weight difference was noted for total body weight (data not shown) or individual tissues between the treated and control groups (Fig. 2a). Gross observations at necropsy showed that beginning at week 1, all mice treated with AuNP had livers that were dark and discolored throughout all lobes (Fig. 2d). This discoloration was also apparent in all spleens by week 8 (Fig. 2d). No significant differences were observed in serum chemistry or cytokine analysis between the treated and control groups (data not shown). Histopathology noted an increased accumulation of AuNPs in Kupffer cells. No other major histopathological findings were noted (Additional file 1: Table S2).

#### AgNP

Mice were dosed once per week for 8 weeks with 5 mg/kg AgNPs based upon stock solution concentration as determined by TGA. No major weight difference was noted for total body weight or individual tissues, with the exception of the spleen. Spleen weights were significantly heavier in the treated group as compared to the control group starting at the first dose of AgNPs (Fig. 2b). No significant differences were observed in serum chemistry or cytokine analysis between the treated and control groups (data not shown). Histopathology noted no apparent difference between the treated and control groups. Particles were not observed in the treated group, and so were not incorporated within the histopathology analysis (Additional file 1: Table S3).

#### SiO_2_ NP

Mice were dosed once per week for 8 weeks with 5 mg/kg SiO_2_NPs. Starting at week 6, three of the SiO_2_ NP treated mice exhibited barbering behavior, as evidenced by the example in Fig. 2e. Although no major weight difference was observed for total body weight, both the liver and the spleens weights were significantly greater than that of the control animals by week 4 and 6, respectively (Fig. 2c).

Beginning at week 3, SiO_2_ NP treated animals had a slightly greater mean histopathology severity score for focal inflammation in the liver than control animals (1.0 ± 0 control vs 2.0 ± 0 treated). The inflammation foci were scattered diffusely throughout the liver and were comprised of small clusters of neutrophils, macrophages, and lymphocytes. Occasionally macrophages presented vesicular cytoplasm. This was the only finding in the entire study with more than occasional 1+ scores. Particles were not observed in the treated group, and so were not incorporated within the histopathology analysis (Additional file 1: Table S4).

**Fig. 3:**
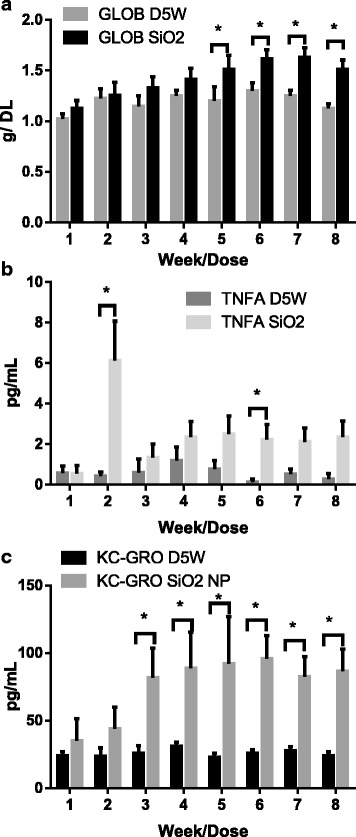
Serum chemistry and cytokine analysis for SiO2 NPs. **a** Serum globulin levels. **b** TNF-α levels. **c** KC-GRO levels. Error bars are standard deviation

Globulin was significantly increased for SiO_2_ NP treated animals as compared to the corresponding controls starting at week 5. TNF-alpha and KC-GRO were also significantly elevated over the control group starting at week 2 and 3, respectively (Fig. 3).

#### Repeat dosing NP accumulation

**Fig. 4:**
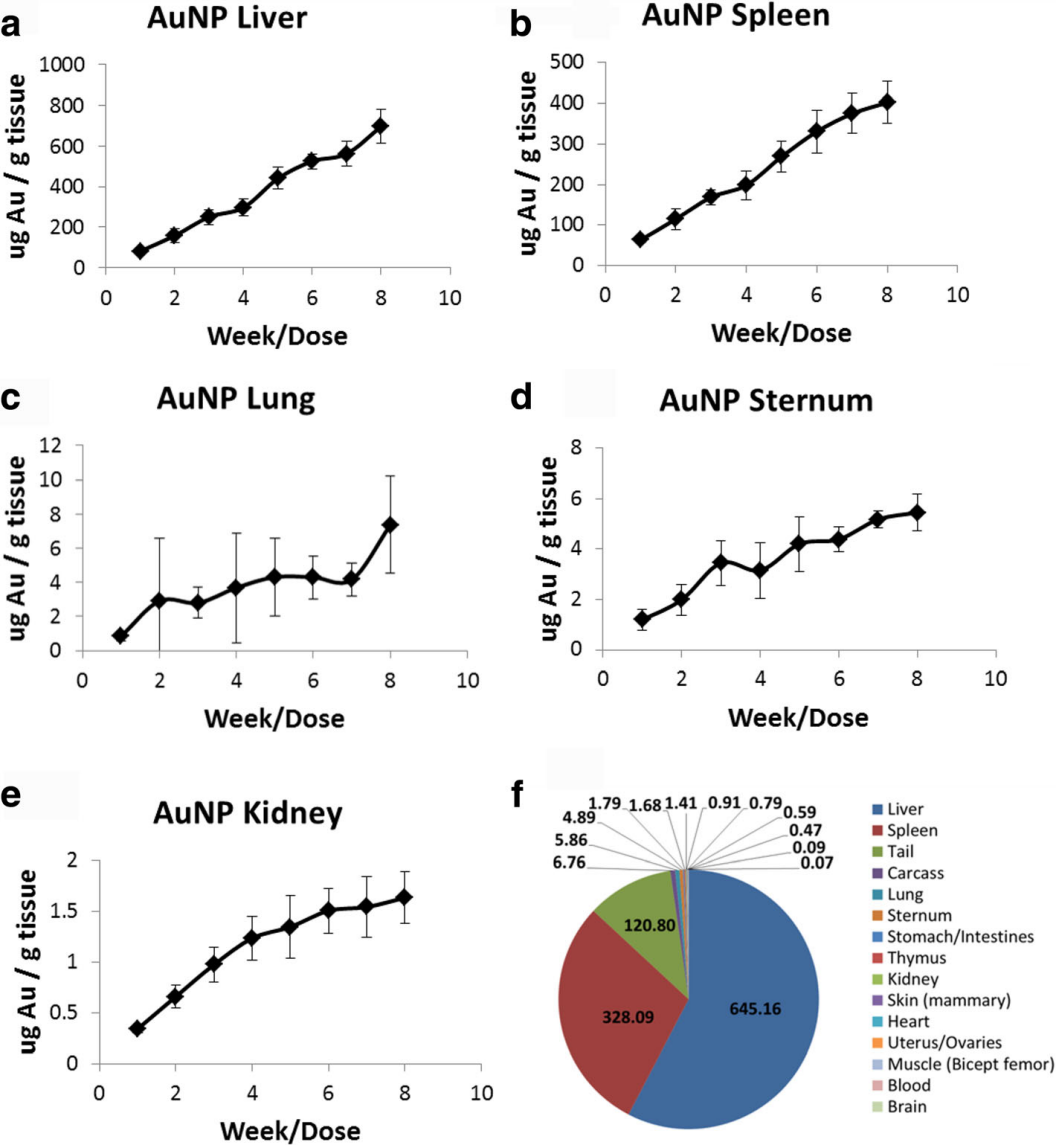
Amount of Au per g tissue for mice dosed with AuNPs 1X per week for 8 weeks. **a** Liver, **b** Spleen, **c** Lung, **d** Sternum, **e** Kidney, **f** Distribution of Au after 8 weeks of dosing. The legend shows the order of AuNP accumulation as measured by μg Au/g tissue

Accumulation of AuNP within organs appeared in the following order for μg Au/g tissue: Liver, spleen, lung, sternum, and kidney (Fig. 4). When repeating this analysis with the animals that had additional tissues removed, after the 8th dose, the order of accumulation was similar, with the stomach/intestine and thymus showing slightly more Au/g tissue than the kidney. The accumulation of AuNP within the other tissues of this larger sample set (skin, heart, uterus, muscle, blood, brain) was minimal, although detectable. Roughly 50% or greater of the injected dose of Au was present within the animal after 8 weeks. No Au signal was detected for the tissues of the control animals.

**Fig. 5:**
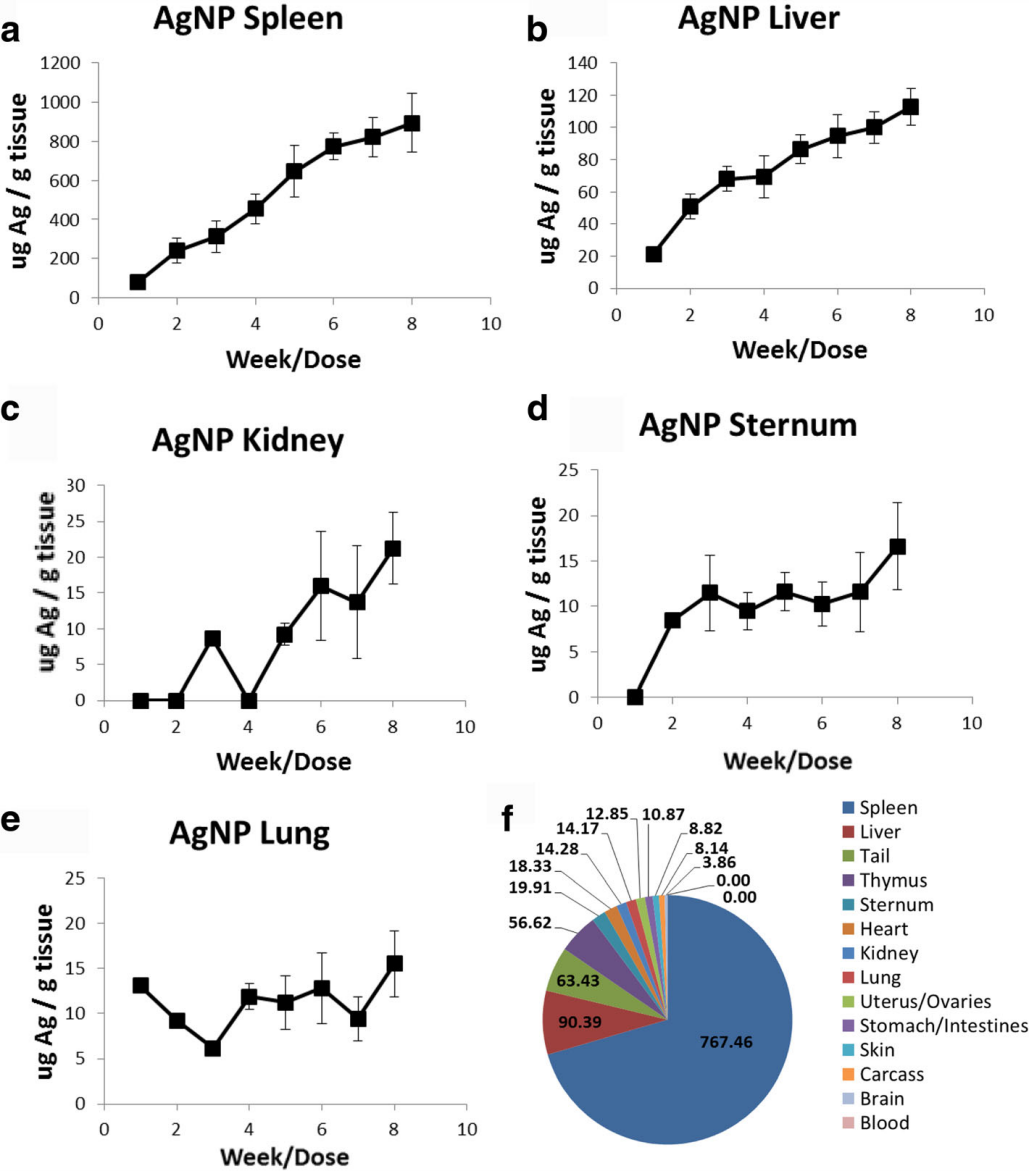
Amount of Ag per g tissue for mice dosed with AgNPs 1X per week for 8 weeks. **a** Spleen, **b** Liver, **c** Kidney, **d** Sternum, **e** Lung **f** Distribution of Ag after 8 weeks of dosing. The legend shows the order of AgNP accumulation as measured by μg Ag/g tissue

Accumulation of AgNPs within organs appeared in the following order for μg Ag/g tissue: Spleen, liver, kidney, sternum, and lung (Fig. 5). With the more extensive organ examination after eight AgNP doses, the order of accumulation was slightly different, with the thymus and heart showing significant levels of Ag. No Ag was detected in either the blood or the muscle. Roughly 50% or greater of the injected dose of Ag was present within the animal after 8 weeks. The majority of the control tissues did not have detectable levels of Ag. A small amount of Ag was inconsistently detected from the tissues of the control group. No pattern of accumulation was able to be distinguished from the Ag signals arising from the control tissues and was not considered incidental to treatment.

Silicon levels in treated animals were not above baseline of the control tissues for ICPMS.

#### Repeat dosing splenocyte changes

**Fig. 6:**
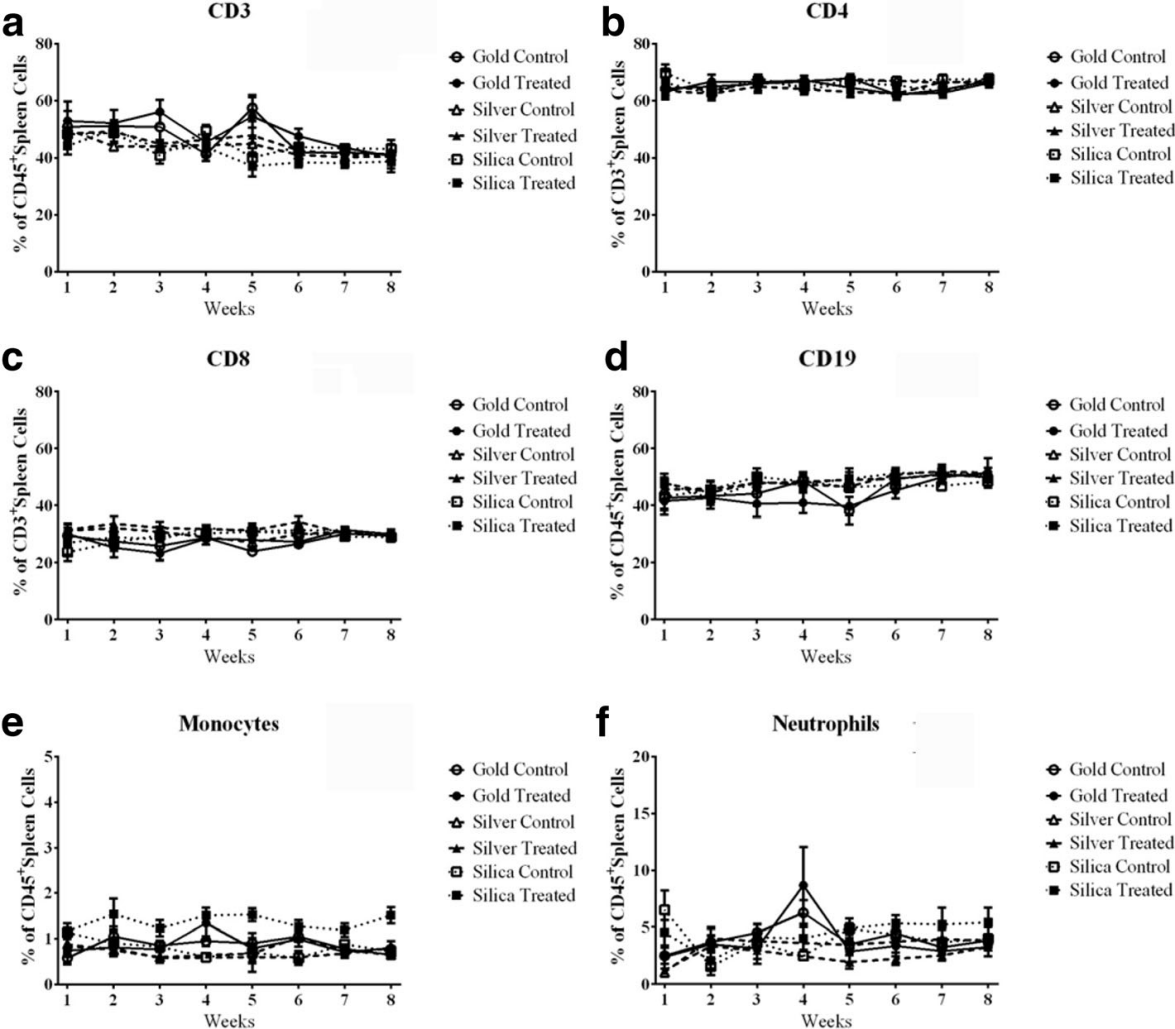
Splenocyte populations as percent of all CD45+ leukocytes. **a** Total T-cells, **b** CD4+ T-Cells, **c** CD8+ T-Cells, **d** Total B-cells, **e** Total macrophages **f** Neutrophils

The flow cytometric analysis of spleen cell populations is shown in Fig. 6. Total T-cells are shown in Fig. 6a and show no treatment related effect over the time course of the study. The major T-cell subpopulations of CD4 and CD8 cells are depicted in Fig. 6b, c and show a similar lack of change. B-cell population numbers are shown in Fig. 6d and are also unremarkable. The populations of monocytes and neutrophils are shown in Fig. 6e, f, respectively. Again no treatment related effects are observed.

## Discussion

Gold, silver, and silica nanoparticles were evaluated based upon their relevance and prevalence in medical applications for both CDER and CDRH [16, 17]. Tissue macrophages have phagocytosis and sequestration of particles as a primary task, and NP durability (e.g. remaining in the particulate state while accumulating within MPS tissues) within mouse models has been previously demonstrated for Au, Ag, and SiO_2_ NPs [9, 18, 19].

The overall conclusion of the study is that the repeated dosing with durable NPs did not reach steady state in any of the MPS of the tissues evaluated as measured by total metal concentration. The majority of the NPs localized in the MPS of the spleen, liver, and lung, with trace amounts of NPs appearing in other tissues (probably also associated with the MPS). Figures 4 and 5 show the linear increase of NPs within the major MPS organs which have a high amount of resident macrophages. The linearity demonstrated throughout the course of the dosing study indicates that the tissues had not reached their limits for sequestration of durable NPs. In addition to the lack of steady state of the MPS within these tissues, AuNPs and AgNPs did not elicit any overt toxicity response over the course of the eight week study. SiO_2_ NP-treated mice began exhibiting SiO_2_-related toxicity starting at week 3 as evidenced by histopathology and an increase in serum pro-inflammatory cytokines.

Dose concentration, time points, repetitions, and NP size were chosen to be similar to the use expected to be seen in clinic. As such, all dose concentrations were chosen that showed a lack of toxicity at a single acute or repeat dosing range [9, 18] (and internal unpublished work). Frequency of dosing as well as the length of the study was also based upon what could reasonably be expected to be seen in clinical use (data based upon the historical submissions of NP products submitted to FDA for review).

### AuNPs

For animals treated with AuNPs, there was a steady increase in the NP load/tissue during the course of the study. No saturation appeared in the liver or spleen, or any other tissue tested. As expected, AuNPs primarily distributed to the liver and spleen. Tails and carcasses analyzed from NP-treated animals also had high NP/tissue loads. The high amount of AuNPs in the tail most likely is due to the multiple injections needed for the study, as well as incomplete dosing into the tail vein. The high carcass load is likely due to the additional MPS tissues not removed (lymph nodes, etc.) which would be expected to engulf the AuNPs as well as the remaining bone marrow residing in the uncollected skeleton. Despite the high tissue load of NPs in the tail, a significant portion of the AuNPs still was available systemically. AuNP showed high variability in both the lung and sternum, possibly due to the low Au load in these tissues.

There have been studies for AuNP biodistribution/bioaccumulation [20, 21], and a few for citrate-capped AuNPs after intravenous injection for rodent models. For example, De Jong et al. showed the same order of organ accumulation for 10 nm AuNPs in a rat model after a single acute injection, with the exception of the thymus, which is listed below the kidneys for the De Jong study, and above the kidneys for the present study [6]. This discrepancy is not considered significant due to the small amount of Au that accumulates in both organs, and accumulates within error on a per g tissue basis (1.7 versus 1.4 for μg/g tissue thymus and kidney, respectively). A second study with 20 nm citrate-capped AuNPs in a rat model showed a biodistribution order of liver, spleen, lung, heart, stomach, thymus, kidney, again with the lower accumulation organs showing some changes in order (in this case the heart) [11]. In contrast, another study by Sonavane et al. showed a much different bioaccumulation order in mice of liver, lung, kidney, spleen, brain, stomach, and heart [7]. It is unclear why there is such a high difference in bioaccumulation for this study, although the dose of 1 g/kg, which is two orders of magnitude over the present study, may be factor. In addition, the high amount of AuNPs within the lung tissue may indicate agglomeration of the NPs, as demonstrated in a previous study [9].

AuNPs treated mice showed no signs of toxicity. However, AuNP-treated animals showed discoloration of the livers after one week of dosing and spleens by week eight as seen in Fig. 2e. Discoloration of the liver and spleen is thought to be due to AuNP accumulation within those tissues. Liver and spleen had the highest accumulation of AuNPs (per gram tissue basis), and so these tissues would be expected to first exhibit the purple discoloration that occurs when AuNPs accumulate in tissues. This discoloration has frequently been observed by other groups investigating AuNP biodistribution [22]. The reported toxicity of AuNPs varies significantly in the literature, ranging from lethal [23] to nontoxic [24]. The wide range of toxicity responses may be due, in part to different preparations, surface coatings, model systems, poorly characterized NPs, impure and/or contaminated dosing solutions. Other groups have previously commented on the difficulty in establishing toxicity studies for NPs [25, 26]. The AuNPs used in the current study were well characterized and had a low endotoxin burden, thus minimizing the chances of external toxicities.

### AgNPs

There was a steady increase in the AgNP load for all tissues evaluated with the exception of the lung and kidney tissues, which were highly variable. As with the AuNPs, NP saturation limits did not appear to be reached in the liver or spleen. Tissues with a small amount of Ag accumulation (such as lung and kidney) showed inconsistent uptake, which may be due to the level of detection of Ag (level of detection ~1 ppm, matrix dependent).

Organ bioaccumulation order was different than the AuNPs, with spleen having the highest NP load/g tissue. This difference may, in part, be due to the AgNPs being five times larger than the AuNPs. In addition to the highest AgNP load, AgNP-treated animals also had significantly heavier spleens starting at the first dose of NPs. The weight difference between the control and AgNP-treated spleens is ~0.02 g (~20 mg). The average dose of AgNP per animals was ~0.0001 g (0.1 mg), so the increase in spleen weight cannot be attributed to simple weight accumulation of the AgNP within the spleen. It has previously been reported in a repeat dose study for AgNPs in rats that there was a significant increase in T and B cells, while the relative cell numbers remained the same. This increase in cells was thought to contribute to the higher spleen weights compared to the control [27]. In this study, no changes in the relative proportions on any major leukocyte population were observed. As with the AuNPs, there were no observed toxicities associated with any of the AgNP doses.

### SiO_2_NPs

Silicon was detected in the tissues of both treated and untreated mice due, in part, to the high baseline levels of silica found in the environment [28–30]. With the exception of week 8 liver, silica was not found over the baseline of any treated tissue. While Si can be readily quanitified (~12 ppb, matrix dependent), it is difficult to establish an acceptable baseline due to the abundance of Si naturally found in the environment and the vast variation and animal specific accumulation of silica within the focal tissues. To help mitigate some of these issues, ICP-MS, being more sensitive to Si levels than NAA, was chosen to monitor Si levels within tissues. Extensive variation in silica levels was observed within several individual animals prohibiting any sample from being rejected from the total data set. Ultimately, Si from the NPs could not be distinguished from Si inherently present within the tissues. Post-study analysis indicated that the high level of Si found within the control animals was, in part, due to the variety of rodent chow used during the study. This finding highlights the important of exercising control on all aspects of study design.

Serum chemistry cytokine levels and increased histopathology scores were noted for the SiO_2_ NPs starting at week 3 with focal liver inflammation and increased circulating pro-inflammatory cytokines. However, no changes in the splenocyte population were observed. It has been demonstrated in previous literature that different silica structures and processing conditions may cause different levels of toxicities [31]. As endotoxin or NP impurities were not present (as determined by the endotoxin assay performed prior to dosing), and given the pattern and location of the toxicity, the toxicity is most likely arising from the nanoparticles within the liver. Despite the lack of accumulation data with this NP, the toxicity that emerged by week three would most likely limit the use of this NP in a repeat-dosing situation as opposed to MPS saturation.

## Conclusions

Despite the increased use of durable nanoparticles in the US market, there is limited understanding on the impact of the accumulation of these particles within the mononuclear phagocytic system. No NP investigated in this study showed a saturation of the MPS within the liver and spleen, with the dosing parameters. Of the three NPs tested, only the silica NPs showed any indication of toxicity. No major changes within the splenocyte population were observed for any of the NPs. The results indicate that the MPS can handle the amounts of durable NP challenges used in this study by sequestering the NPs without significant impact on the structure or function of the tissues analyzed. Additional work is ongoing to investigate the impact and secondary effects if any of durable NP bioaccumulation.

## Additional file

Additional file 1: Figure S1. Schematic of experimental design. Note NP treatment is for each of the three NPs tested (e.g. 56 animals for AuNPs, 56 animals for AgNPs, 56 animals for SiO_2_ NPs). Note that this scheme does not take into account the extra animals that were dosed to allow for sufficient animal numbers at the end of the study (e.g. due to mis-dosing or animal death). **Figure S2.** DLS stability evaluation of NP dosing solutions. NPs were dispersed into D5W at representative dosing concentrations. DLS histograms were recorded up to 72 h after initial dilution. Representative histograms for **A)** AuNPs **B)** AgNPs **C)** SiO_2_ NPs. Dosing took no longer than 3.5 h for any NP. **Table S1.** Staining template for splenocyte analysis. **Table S2.** Summary Incidence Tables for AuNPs. **Table S2.** Summary Incidence Tables for AgNPs. **Table S4.** Summary Incidence Tables for SiO_2_NPs. (DOC 664 kb)

## Abbreviations

AgNPs: Silver nanoparticles
AuNPs: Gold nanoparticles
CDER: Center for Drug Evaluation and Research
CDRH: Center for Devices and Radiological Health
D5W: 5% dextrose in water
DLS: Dynamic light scattering
DPBS: Dulbecco’s Phosphate Buffer Solution
ICP-MS: Inductively coupled plasma mass spectrometry
LAL: Limulus Amebocytes Lysate
MPS: Mononuclear phagocyte system
NAA: Neutron activation analysis
NPs: Nanoparticles
PIXE: Proton induced X-ray emission (LAL)
RT: Room temperature
SiO_2_ NPs: Silica NPs
TEM: Transmission electron microscopy
TGA: Thermal gravimetric analysis
US FDA: United States Food and Drug Administration
UV–vis: Ultraviolet–visible
