# Supplementary material for: Evaluating the potential of gold, silver, and silica nanoparticles to saturate mononuclear phagocytic system tissues under repeat dosing conditions
Source: Part Fibre Toxicol. 2017 Jul 17;14:25. doi: 10.1186/s12989-017-0206-4 (PMC5513057; doi:10.1186/s12989-017-0206-4)
Supplement: Additional file 1: Figure S1. — Schematic of experimental design. Note NP treatment is for each of the three NPs tested (e.g. 56 animals for AuNPs, 56 animals for AgNPs, 56 animals for SiO2 NPs). Note that this scheme does not take into account the extra animals that were dosed to allow for sufficient animal numbers at the end of the study (e.g. due to mis-dosing or animal death). Figure S2. DLS stability evaluation of NP dosing solutions. NPs were dispersed into D5W at representative dosing concentrations. DLS histograms were recorded up to 72 h after initial dilution. Representative histograms for A) AuNPs B) AgNPs C) SiO2 NPs. Dosing took no longer than 3.5 h for any NP. Table S1. Staining template for splenocyte analysis. Table S2. Summary Incidence Tables for AuNPs. Table S2. Summary Incidence Tables for AgNPs. Table S4. Summary Incidence Tables for SiO2NPs. (DOC 664 kb) [file 12989_2017_206_MOESM1_ESM.doc]

**
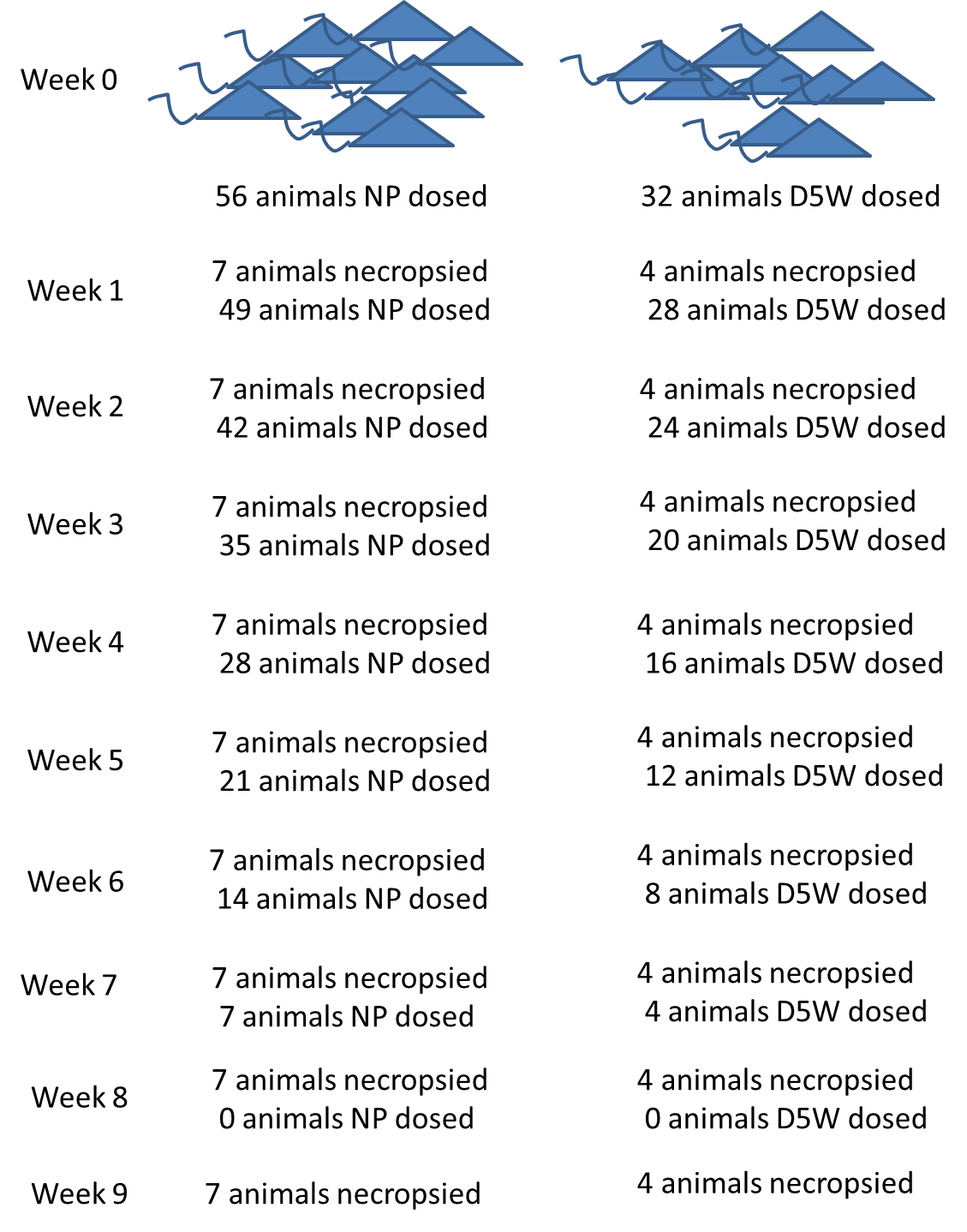
**

**Supplemental Figure 1**: Schematic of experimental design. Note NP treatment is for each of the three NPs tested (e.g. 56 animals for AuNPs, 56 animals for AgNPs, 56 animals for SiO_2_ NPs). Note that this scheme does not take into account the extra animals that were dosed to allow for sufficient animal numbers at the end of the study (e.g. due to mis-dosing or animal death).


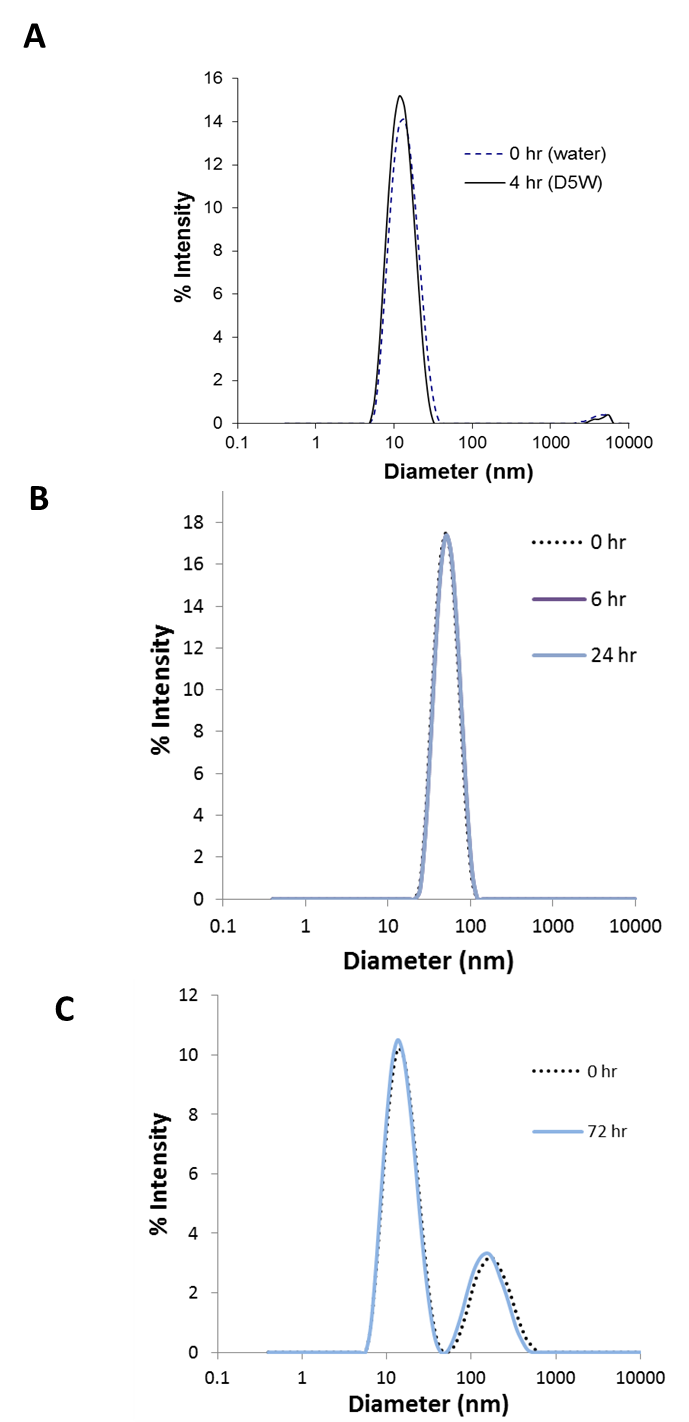


**Supplemental Figure 2 .** DLS stability evaluation of NP dosing solutions. NPs were dispersed into D5W at representative dosing concentrations. DLS histograms were recorded up to 72 hour after initial dilution. Representative histograms for **A)** AuNPs **B)** AgNPs **C)** SiO_2_ NPs. Dosing took no longer than 3.5 hours for any NP.

**Supplementary Table I.** Staining template for splenocyte analysis

Pathology Incidence Tables:

Numbers in parentheses for each organ represent the number of animals examined in each experimental group. The number in each entry represents the number of animals in that group that were found to have that specific pathological finding. Note that some descriptions of individual pathology terms run onto two lines.

**Supplementary Table II.** Summary Incidence Tables for AuNPs

**Supplementary Table III.** Summary Incidence Tables for AgNPs

**Supplementary Table IV.** Summary Incidence Tables for SiO_2_NPs
